# Therapeutic approaches for pulmonary artery pseudoaneurysms and analysis of outcomes

**DOI:** 10.1007/s00330-025-11749-0

**Published:** 2025-06-14

**Authors:** Serhat Akis, Young Ho So, Junyoung Lee, Kwang Nam Jin, Ye Ra Choi

**Affiliations:** 1 Department of Radiology, Dokuz Eylul University Medical School Hospital, Izmir, Türkiye; 2 Department of Radiology, Seoul Metropolitan Government Seoul National University Boramae Medical Center, Seoul, Republic of Korea; 3 Department of Radiology, Seoul National University College of Medicine, Seoul, Republic of Korea; 4 Institute of Radiation Medicine, Seoul National University Medical Research Center, Seoul, Republic of Korea

**Keywords:** Pulmonary artery, Pseudoaneurysm, Bronchial artery, Embolization, Hemoptysis

## Abstract

**Objectives:**

To evaluate the therapeutic approaches for pulmonary artery pseudoaneurysms (PAPs) with various etiologies and types, and outcomes.

**Materials and methods:**

Between March 2010 and March 2024, 30 PAPs were identified in 29 patients. We analyzed the medical records of 29 PAPs in 28 patients whose etiologies were confirmed. Patient characteristics, underlying lung abnormalities, PAPs’ characteristics, therapeutic approaches, and outcomes were evaluated.

**Results:**

Twenty-nine PAPs were treated in 28 patients (mean age 59 years ± 12.4; 25 males). The etiologies of PAPs were pulmonary tuberculosis (*n* = 22), necrotizing pneumonia (*n* = 6), and iatrogenic (*n* = 1). In one tuberculosis patient, two PAPs occurred at different times. Hemoptysis volume varied according to etiology, with no hemoptysis in iatrogenic PAP. In 21 PAPs, pulmonary artery embolization (PAE) (*n* = 15), embolization via systemic-to-pulmonary shunt or bronchial/non-bronchial systemic artery embolization (BAE/SAE) (*n* = 5), and medical treatment (*n* = 1) were performed after pulmonary arteriography. For eight PAPs, embolization via systemic-to-pulmonary shunt or BAE/SAE (*n* = 7), and medical treatment (*n* = 1) were performed without additional pulmonary arteriography. Two patients underwent medical treatment because they had small (< 5 mm) or isolated PAP from the pulmonary artery. In 23 patients (24 PAPs), PAPs regressed or hemoptysis ceased. Two patients underwent surgery during follow-up. Three patients died from sepsis or massive hemoptysis. There were no complications related to the procedure.

**Conclusion:**

PAE is an effective treatment for PAPs, but empirical embolization via systemic-to-pulmonary artery shunt can be an effective alternative treatment for PAPs not detected on pulmonary angiography. In addition, medical treatment can be considered in small or isolated PAPs.

**Key Points:**

***Question*** *Due to the rarity of pulmonary pseudoaneurysms, mostly seen in severe chronic infectious pulmonary disease such as tuberculosis, percutaneous treatment has not yet been standardized.*

***Findings*** *Transcatheter embolization via the pulmonary artery itself and often via systemic artery, in particular bronchial-to-pulmonary-artery shunts, is an effective treatment of those pseudoaneurysms.*

***Clinical relevance*** *Life-threatening pulmonary hemorrhage can be treated using superselective embolization which requires an individual treatment plan based on the etiology of hemorrhage, the clinical status of the patient, and the vascularization of the pseudoaneurysms as shown by CT and angiography.*

**Graphical Abstract:**

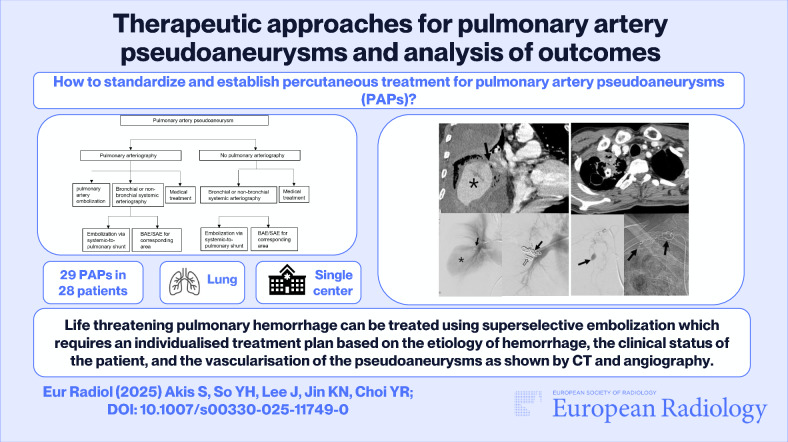

## Introduction

A pulmonary artery pseudoaneurysm (PAP) is an uncommon disease entity that leads to hemoptysis, with a prevalence of 5–11% in patients who have undergone embolization of bronchial and non-bronchial systemic collateral arteries [1–3]. PAP has a diverse etiology, including trauma, malignancy, bronchiectasis, lung abscess, Behcet’s disease, and other acute or chronic inflammatory lung diseases [1–4]. Recently, coronavirus disease 2019 (COVID-19) has been listed as one of the causes of PAPs since its pandemic period [5–7].

The symptoms and amount of hemoptysis in patients with PAP tend to vary according to the underlying etiology and type [8]. When the PAP is unruptured or non-communicating with the airway, the patient may not experience hemoptysis or only exhibit blood-tinged sputum. However, in majority of cases, PAP can cause life-threatening massive hemoptysis when it is not properly treated. Therefore, the detection and appropriate management are quite important for patients with hemoptysis related to PAPs. Currently, multidetector computed tomography (MDCT) is the mainstay for detection and provides substantial information regarding the etiology and vascular anatomy, which are essential for establishing a treatment plan.

Various treatment options can be considered to treat PAPs according to the etiology, type, location, and clinical presentation, including surgical resection, endovascular embolization, or medical treatment [9–13]. Among these, the surgical option often has limitations in patients with massive hemoptysis and has been associated with high morbidity and mortality. In terms of endovascular treatment, embolization of any feeding pulmonary arterial branch is essential for successful hemostasis. However, according to a previous study, PAPs are not always visualized on pulmonary arteriography and, occasionally, only visualized on bronchial or non-bronchial systemic arteriography [12]. As another option, medical treatment can be adopted in small infectious PAPs [13]. In this respect, various therapeutic approaches should be considered for the successful treatment of hemoptysis originating from PAP, and individualized treatment plans should be established according to the clinical presentation.

Therefore, the objective of the current study was to evaluate therapeutic approaches for PAPs of various etiologies and types and their outcomes.

## Materials and methods

This study was a retrospective study, which was approved by the institutional review board, and the requirement of informed consent was waived.

### Patients

**Fig. 1:**
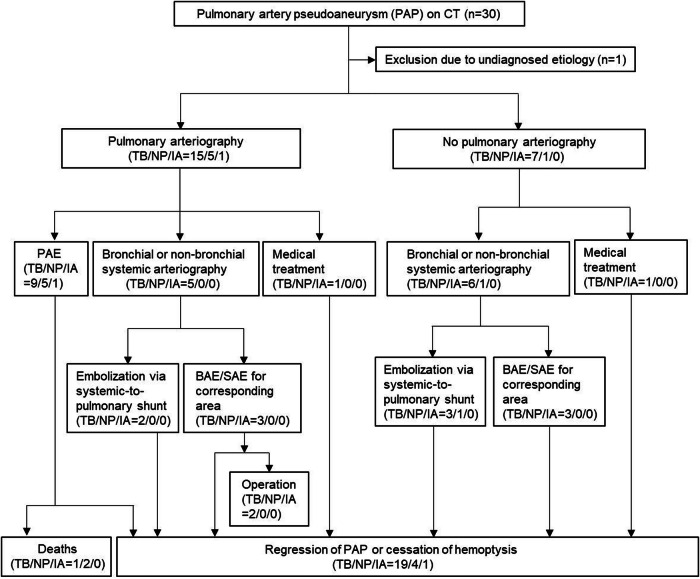
Flowchart of the treatment of pulmonary artery pseudoaneurysms (PAPs) and outcomes. CT, computed tomography; TB, tuberculosis; NP, necrotizing pneumonia; IA, iatrogenic injury; PAE, pulmonary artery embolization; BAE/SAE, bronchial/non-bronchial systemic artery embolization

Between March 2010 and March 2024, 5962 consecutive patients underwent thoracic computed tomography (CT) angiography for the evaluation of bleeding, which was read by four chest radiologists. For the CT scans diagnosed with PAP, two radiologists (an interventional and a chest radiologist with 7 and 17 years of clinical experience, respectively) reviewed and excluded the cases misinterpreted as PAP in consensus. A total of 30 PAPs were identified in 29 patients, and this retrospective study was conducted on 29 PAPs in 28 patients (mean age 59 years ± 12.4; 25 males) in whom the etiology of the PAP was confirmed (Fig. 1).

Electronic medical records were reviewed to evaluate patients’ age, sex, past medical history, laboratory findings, clinical symptoms, treatment methods, follow-up periods, and outcomes. CT findings were analyzed by means of axial, multiplanar reformation (MPR), and maximum intensity projection (MIP) images to evaluate the etiologies and characteristics of PAPs; size, location, systemic artery hypertrophy, and the continuity between the PAP and pulmonary artery (Table 1). CT scans were obtained on 16- to 64-multidetector CT scanners (Brilliance 64, Philips; Ingenuity Elite 64, Philips; LightSpeed Pro 16, GE Healthcare; or Somatom sensation 64, Siemens Healthcare). The CT scan parameters were as follows: 100–120 kVP, 86–458 mA, and 1–4 mm thickness.

**Table 1:** Basic characteristics of patients and pulmonary artery pseudoaneurysms (PAPs)

Parameters	Values
Number of PAPs/patients	29/28 (M:F = 25:3)
Patient’s age at treatment (years)	42–89 (mean 59 ± 12.4)
Etiology of PAP	
Tuberculosis	22/29 (76%)
Necrotizing pneumonia	6/29 (21%)
Iatrogenic injury	1/29 (3%)
Location of PAPs	
Right (RUL/RML/RLL)	11/1/5
Left (LUL/LLL)	9/3
Size of the PAPs (mm)	10.9 ± 11.3
Bronchial or non-bronchial systemic artery hypertrophy	
Tuberculosis	22/22 (100%)
Necrotizing pneumonia	3/6 (50%)
Iatrogenic injury	0/1 (0%)
Previous BAE/SAE history	10/29 (34%)
Hemoptysis	
Present	28/29 (97%)
None	1/29 (3%)
Coagulopathy	3/29 (10%)

*BAE/SAE* bronchial/non-bronchial systemic artery embolization, *RUL* right upper lobe, *LUL* left upper lobe, *RML* right middle lobe, *RLL* right lower lobe, *LLL* left lower lobe

### Therapeutic approach

The therapeutic approach for PAP was planned based on the underlying etiology and CT and clinical findings (Fig. 1). Pre-evaluation using bronchoscopy was not performed except in one patient because of the potential risk of triggering massive hemoptysis. In general, all patients were systemically administered a hemostatic agent (Tranexamic acid injection; Shinpoong Pharmaceutical). Transcatheter embolization was performed after diagnostic pulmonary arteriography or bronchial or non-bronchial systemic arteriography. Pulmonary arteriography was performed in patients with a PAP attributed to iatrogenic injury or a PAP ≥ 5 mm with continuity to the pulmonary artery. Alternatively, pulmonary arteriography was not performed in patients with a PAP isolated from the pulmonary artery or a PAP < 5 mm. Pulmonary artery embolization (PAE) was performed when the PAP was visualized on the pulmonary arteriography. When the PAP was not visualized on pulmonary arteriography, bronchial or non-bronchial systemic arteriography was performed, followed by embolization via systemic-to-pulmonary artery shunt or bronchial/non-bronchial systemic artery embolization (BAE/SAE) in the area corresponding to the PAP. In patients who did not undergo pulmonary arteriography initially, bronchial or non-bronchial systemic arteriography was performed and followed by embolization via systemic-to-pulmonary artery shunt or BAE/SAE in the area corresponding to the PAP. Among the patients who received PAE, additional BAE/SAE was performed in the presence of bronchial or non-bronchial systemic artery hypertrophy.

### Endovascular procedures

The endovascular approach was initiated from the puncture of a femoral artery for a systemic arterial approach or a femoral vein for a pulmonary arterial approach under ultrasound guidance using a micropuncture set, followed by a 5-F vascular sheath insertion. For PAE, segmental or subsegmental pulmonary arteriography was performed by using 5-F angiographic catheters (Davis catheter; Cook) to identify the feeding pulmonary arterial branch. Upon identifying the feeding branch and PAP, embolization was performed coaxially with a 1.7-F or 2.0-F microcatheter (Progreat; Terumo, Veloute; Asahi Intecc) using 3–6 mm microcoils (Tornado®, Nester®; Cook) and/or a mixture of N-butyl cyanoacrylate (NBCA) (Histoacryl; B. Braun) and lipiodol (Guerbet) (ratio 1:3). For BAE/SAE, arteriography was performed by using 5-F angiographic catheters (GRB catheter; Seongwon Medical, Davis catheter, Cobra catheter; Cook). Selective BAE/SAE was performed with a 1.7 to 2.0-F microcatheter (Progreat; Terumo, Veloute; Asahi Intecc) using 250–500 microns polyvinyl alcohol (PVA) particles (Contour; Boston Scientific) and 560–1000 microns gelatin sponge particles (EGgel S plus, Engain). Embolization of the PAP visualized through systemic-to-pulmonary artery shunt was performed with a 1.7 to 2.0-F microcatheter (Progreat; Terumo, Veloute; Asahi Intecc) using 250–500 microns PVA particles or a mixture of NBCA and lipiodol (ratio 1:3). For all patients, selective arteriography was performed based on the anatomic information from CT scan.

### Definitions and outcome evaluation

Clinical and angiographic findings, procedure-related complications, and the outcomes were evaluated. Patients who showed the following criteria were considered to have coagulopathy: prothrombin time international normalized ratio (PT-INR) < 1.5, activated partial thromboplastin time (aPTT) < 45 s, or platelet count of < 80,000/L [14, 15]. Procedure-related complications were graded according to the Cardiovascular and Interventional Radiological Society of Europe (CIRSE) classification system for complications [16].

## Results

A total of 29 PAPs were treated in 28 patients (M:F = 25:3; age range: 42–89 years). The etiologies of identified PAPs were as follows: pulmonary tuberculosis (*n* = 22), necrotizing pneumonia (*n* = 6), and iatrogenic (*n* = 1). In one patient with tuberculosis, two PAPs occurred at different times. Ten patients (9 tuberculosis and 1 necrotizing pneumonia) had a history of previous BAE/SAE. The mean PAP size was 10.9 mm (range: 3–60 mm). The amount of hemoptysis varied according to the etiology, reaching up to 1000 cc. In one patient with iatrogenic PAP, which was caused by a needle injury to the lung during the bedside thoracentesis, hemoptysis was not observed. On CT images, a total of 25 PAPs displayed continuity with the pulmonary artery, whereas PAP was isolated from the pulmonary artery in the remaining 4 PAPs. PAPs were located in the right upper lobe (11/29, 41.4%), left upper lobe (9/29, 31.0%), right middle lobe (1/29, 3.4%), right lower lobe (5/29, 13.8%), and left lower lobe (3/29, 10.4%), respectively. Among these, PAP was mainly located in the upper lobe in patients with pulmonary tuberculosis (19/22, 86.4%). Bronchial or non-bronchial systemic artery hypertrophy was observed in all patients with pulmonary tuberculosis (22/22) and in three with necrotizing pneumonia (3/6), but was absent in those with iatrogenic pulmonary artery injury (0/1). In one patient, bleeding focus was localized by using bronchoscopy for pre-evaluation. Coagulopathy was observed in three patients (Table 1).

**Fig. 2:**
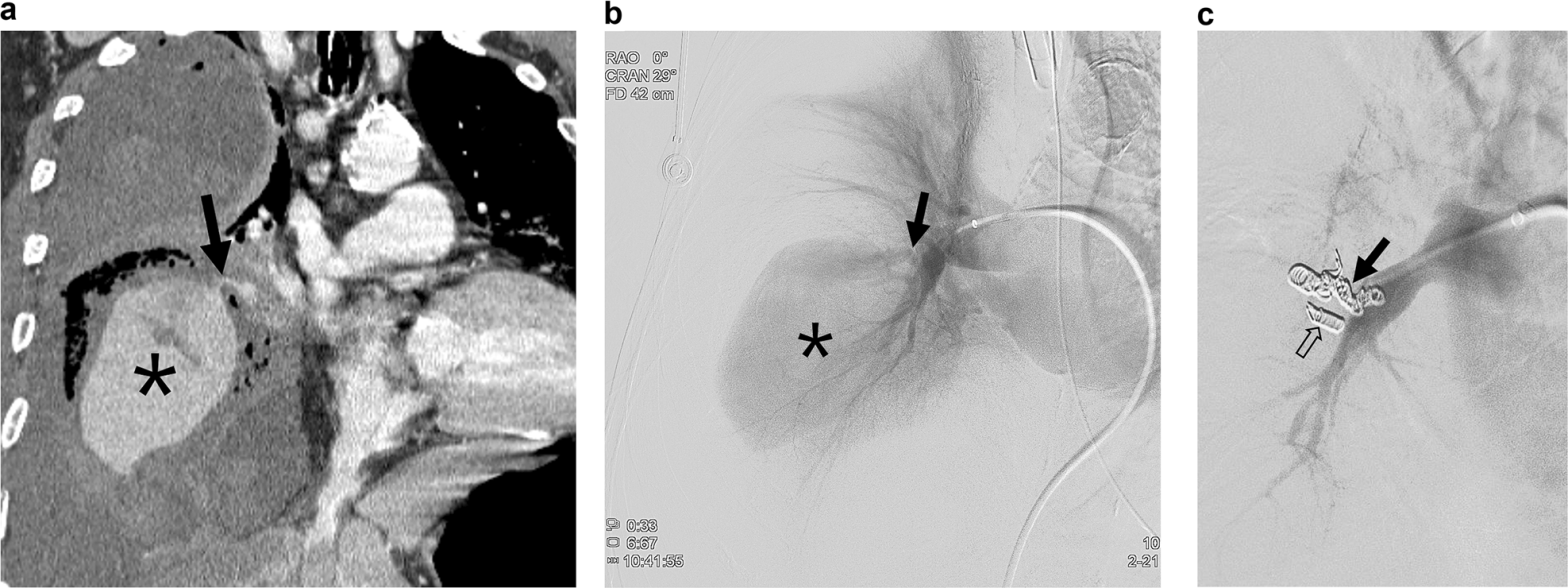
A 78-year-old male was admitted with fever and chills 2 months after radiation therapy for small cell lung cancer in the right lower lobe. **a** On the chest CT, a huge pulmonary artery pseudoaneurysm (PAP) (*) surrounded by necrotic lung tissue, suggestive of necrotizing pneumonia, was identified in the right middle lobe of the lung. The leaking point of the pseudoaneurysm from the pulmonary artery (arrow) can be noticed. **b** Similar to the CT findings, a huge PAP (*) and a short neck of PAP (arrow) can be visualized on the pulmonary arteriography. **c** Embolization of the pseudoaneurysm neck was performed with multiple microcoils (arrow). Complete exclusion of the PAP can be observed, with the filling of PAP no longer visible on the pulmonary arteriography. Some coils appear to have migrated into the PAP (open arrow) or protruded into the pulmonary artery owing to the short pseudoaneurysm neck. However, the patient died from sepsis after 3 weeks

**Fig. 3:**
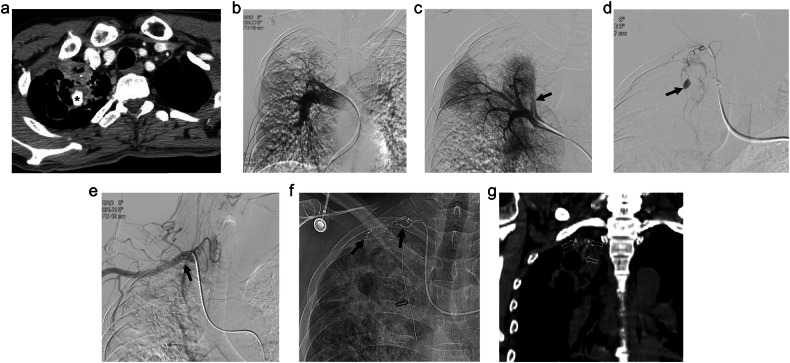
A 47-year-old male was admitted with cough and massive hemoptysis. The patient had a history of pulmonary tuberculosis. **a** The CT scan reveals the presence of a pulmonary artery pseudoaneurysm (PAP) (*) in the right upper lobe of the lung. Surrounding tuberculous cavity and consolidation due to tuberculous pneumonia can also be observed. **b** Pulmonary arteriography was performed using a pigtail catheter; however, the PAP could not be visualized. **c** Upon selective arteriography, the suspected pulmonary arterial branch supplying the PAP was not opacified owing to competitive systemic arterial flow (arrow). **d** Upon suspecting that the PAP received supply from the systemic artery, bronchial and non-bronchial systemic arteriography were performed to evaluate potential collateral arteries. The PAP was visualized via systemic-to-pulmonary artery shunt on the selective arteriography of the branch of the subclavian artery (arrow). **e**, **f** Embolization was performed via systemic-to-pulmonary shunt using a mixture of N-butyl cyanoacrylate (NBCA) and lipiodol (ratio 1:3). Embolic material casted in the main feeding systemic arterial branches of the PAP (arrows). Upon performing postembolic subclavian arteriography, the PAP was not visualized. Microcatheter was located in the internal mammary artery for the additional bronchial/non-bronchial systemic artery embolization (BAE/SAE) on the fluoroscopic image (open arrow). **g** Precontrast CT scan shows embolic material casted in the peripheral pulmonary artery around the PAP (open arrows), which was barely visualized on the fluoroscopic images. After embolization, the patient underwent additional BAE/SAE, and massive hemoptysis stopped

**Fig. 4:**
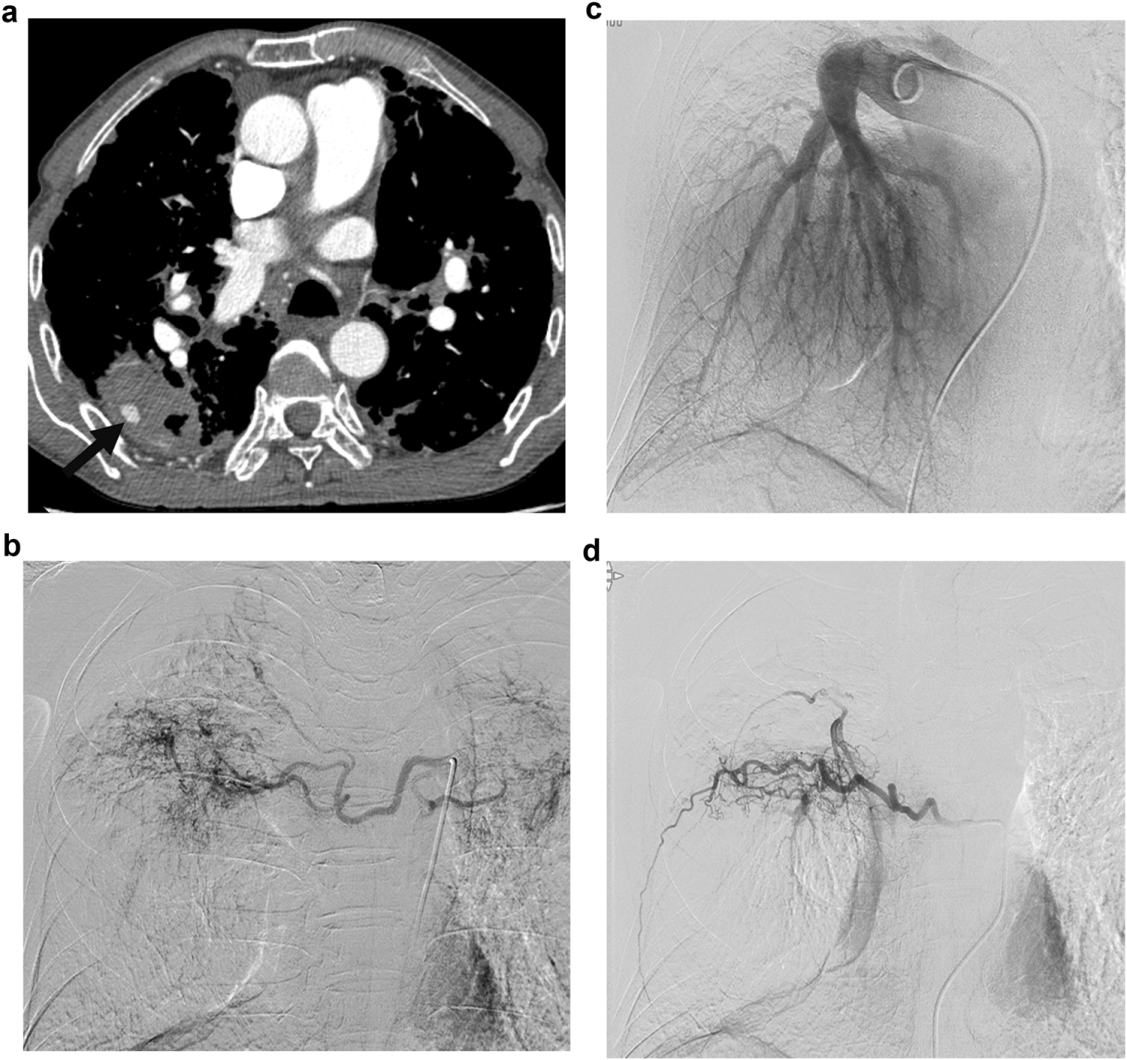
A 77-year-old male was admitted with hemoptysis. **a** CT scan shows a pulmonary artery pseudoaneurysm (PAP) in the right lower lobe. The PAP is located in the tuberculous cavity, surrounded by hematoma, and isolated from the pulmonary arterial branch (arrow). **b** Pulmonary arteriography was performed using a pigtail catheter; however, the PAP could not be visualized. **c**, **d** The PAP was not visualized upon performing bronchial and intercostal arteriography. Empirical bronchial/non-bronchial systemic artery embolization (BAE/SAE) in the area corresponding to the PAP was performed using 355–500 microns PVA particles and hemoptysis stopped after embolization

**Fig. 5:**
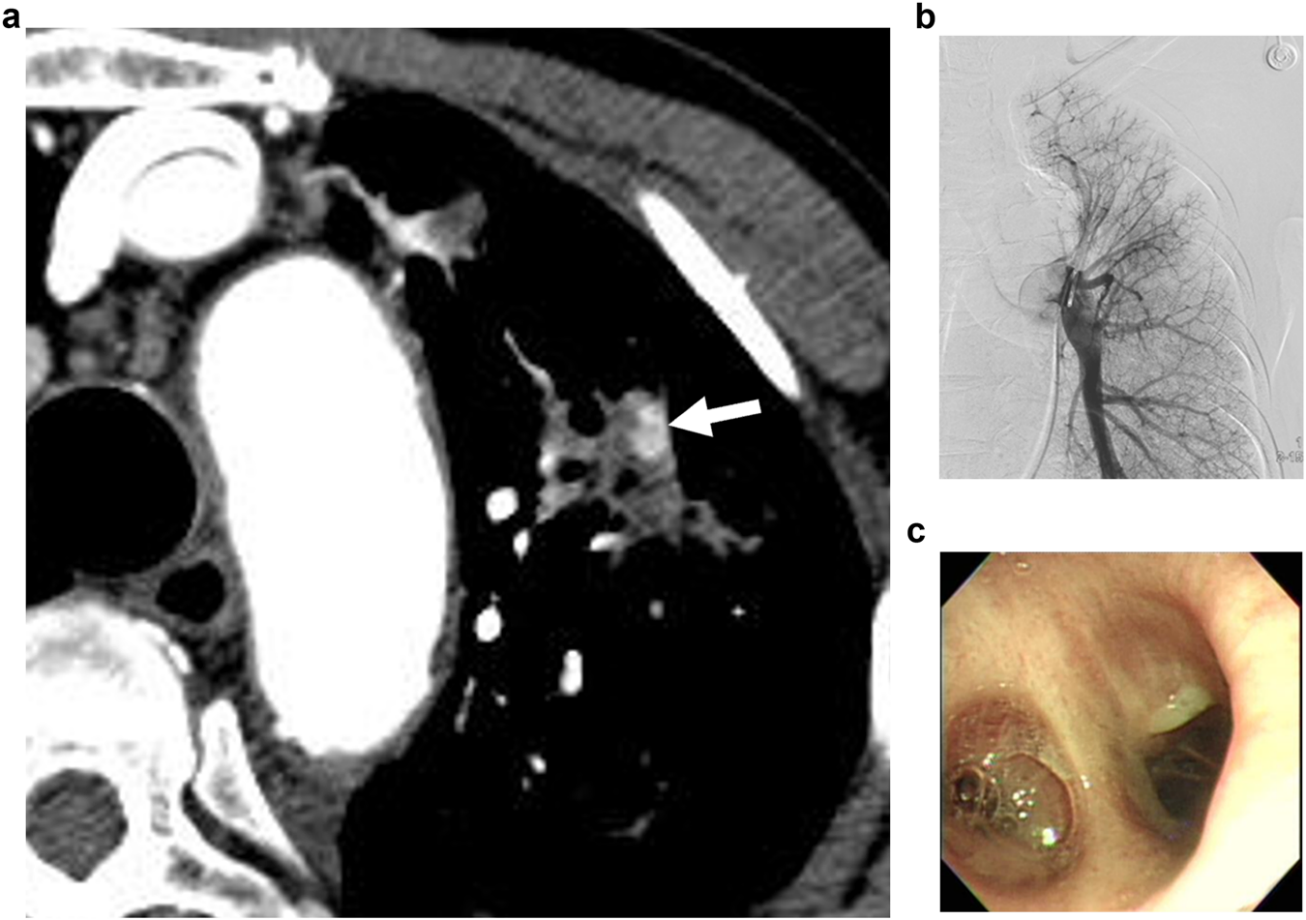
A 72-year-old man with a history of tuberculosis was admitted with blood-tinged sputum. **a** CT angiography shows a pulmonary artery pseudoaneurysm (PAP) in the left upper lobe of the lung (arrow). **b** Pulmonary arteriography was performed using a pigtail catheter. However, the PAP could not be visualized. **c** The patient underwent conservative treatment with tranexamic acid and antitussive administration. After treatment, the patient’s blood-tinged sputum improved. Upon performing a bronchoscopy, no bleeding in the corresponding area was detected

Pulmonary arteriography was performed in 21 PAPs (15 tuberculosis, 5 necrotizing pneumonia, and 1 iatrogenic injury), with PAE performed in 15 PAPs (9 tuberculosis, 5 necrotizing pneumonia, 1 iatrogenic injury) (Fig. 2). Of the 21 PAPs subjected to pulmonary arteriography, 6 PAPs could not be visualized upon performing the procedure. Among these, 5 PAPs were treated with embolization via systemic-to-pulmonary artery shunt (*n* = 2) (Fig. 3) or BAE/SAE in the area corresponding to the PAP (*n* = 3) (Fig. 4). In the remaining one, further endovascular treatment was not performed because hemoptysis ceased after tranexamic acid administration (Fig. 5). Among the patients who underwent PAE, 10 underwent additional BAE/SAE.

Pulmonary arteriography was not performed in 8 PAPs (7 tuberculosis and 1 necrotizing pneumonia). Among these, bronchial or non-bronchial systemic arteriography was performed in 7 (6 tuberculosis, 1 necrotizing pneumonia), followed by embolization via systemic-to-pulmonary artery shunt (*n* = 4) or BAE/SAE in the area corresponding to the PAP (*n* = 3). In the remaining case of tuberculosis, further endovascular treatment was not performed because hemoptysis ceased after tranexamic acid administration.

In three patients who underwent embolization via systemic-to-pulmonary artery shunt, a mixture of NBCA and lipiodol was used as an embolic material.

The mean follow-up period was 59.7 months. Imaging investigations (CT scan in 15 and chest radiography in 13) and bronchoscopy (in 4) were performed during the follow-up period.

Considering 24 PAPs identified in 23 patients, PAP regression or cessation of hemoptysis was achieved. Among these, hemoptysis stopped completely in 13 PAPs of 13 patients (11 tuberculosis and 2 necrotizing pneumonias) and blood-tinged sputum was sustained in 9 PAPs of 8 patients, which disappeared within 1 month (7 tuberculosis and 1 necrotizing pneumonia). In one patient with necrotizing pneumonia due to lung cancer, hemoptysis decreased and the PAP regressed on follow-up CT scan. In one patient with iatrogenic PAP, in whom hemoptysis was not observed, the PAP regressed on follow-up CT scan. Among the patients who underwent PAE, 3 patients (1 tuberculosis and 2 necrotizing pneumonias) died from sepsis or massive hemoptysis (Fig. 2). Two patients with tuberculosis underwent surgical lobectomy in the area corresponding to the PAP owing to persistent hemoptysis (Fig. 1). There were no procedure-related complications.

## Discussion

A pseudoaneurysm is a contained arterial rupture surrounded by relatively thin adventitia or surrounding perivascular soft tissue, while a true aneurysm involves all three layers [17]. PAPs are detected in up to 11% of patients examined for hemoptysis and in approximately 14% of those with chronic pulmonary tuberculosis [3]. A PAP that originates from the tuberculous cavity is known as a Rasmussen aneurysm [18]. Other common causes of PAPs include infection, vasculitis, neoplasm, trauma, and iatrogenic injury [1–4].

To treat PAPs, several reports support the use of endovascular treatment as a first-line treatment whenever possible [3, 8, 17, 19–22]. The percutaneous direct puncture embolization can be selected for PAPs that involve peripheral branches of the pulmonary artery by employing embolizing agents such as thrombin or glue under CT, ultrasound, and/or fluoroscopy guidance [23–25]. Surgical treatment such as pneumonectomy, lobectomy, prosthetic graft or pericardial patch interposition, pulmonary artery ligation, or hilar clamping with direct arterial repair can be performed in certain cases. Medical treatment can be another option, particularly in patients with small infectious PAPs [13, 26, 27].

In our study, treatment options were selected based on the underlying cause of the PAP, the clinical status of the patients, and CT findings. Most of the cases in our study were PAPs secondary to pulmonary tuberculosis, which showed a variable clinical status and CT findings according to the underlying lung parenchymal involvement. Among a total of 22 PAPs of pulmonary tuberculosis, we performed PAE after pulmonary arteriography in 9 cases. Unlike PAPs due to other causes, pulmonary tuberculosis commonly exhibits systemic-to-pulmonary artery shunt, and we performed embolization via this shunt or BAE/SAE in the area corresponding to the PAP with or without pulmonary arteriography. Torikai et al performed successful embolization of PAPs through systemic-to-pulmonary artery shunt in 5 patients [28]. In the current study, most cases were tuberculosis-related PAPs, and dilated and tortuous bronchial and non-bronchial systemic artery hypertrophy was observed, along with systemic-to-pulmonary artery shunt. Shin et al described the four types of PAPs based on the angiographic findings [12]. The authors treated PAPs, classified into type C, and visualized only upon bronchial and non-bronchial systemic arteriography by embolizing bronchial and non-bronchial systemic arteries. Considering our cases and those reported previously, although PAE is a direct and effective treatment for PAPs originating from pulmonary tuberculosis, embolization via systemic-to-pulmonary artery shunt or BAE/SAE in the area corresponding to the PAP can also be a crucial treatment option.

In some patients with chronic tuberculosis, small peripherally located PAPs occurred with surrounding consolidation. Among these, we noted the cessation of hemoptysis in two patients after medical treatment with a hemostatic agent and without PAE or BAE/SAE. These patients exhibited stable vital signs without coagulopathy at the time of admission. One patient underwent pulmonary arteriography, but the PAP was not visualized; the other did not undergo any diagnostic arteriography. Unlike systemic circulation, the pulmonary artery is not exposed to high arterial pressure, which allows contained injuries to heal without a high risk of rupture of the PAP [29]. Accordingly, conservative medical treatment, including the administration of a hemostatic agent, plays a crucial role and can be another option in itself to treat PAP in non-urgent patients exhibiting a stable clinical condition without coagulopathy.

The therapeutic approaches for PAPs vary according to their etiology and the duration of the underlying disease. Considering our cases, we identified seven PAPs of non-tuberculous etiology, including six necrotizing pneumonias and one iatrogenic injury. In the case of necrotizing pneumonia, bronchial or non-bronchial systemic artery hypertrophy was present or absent according to the degree of chronicity of the underlying disease. For instance, we performed additional BAE/SAE to control hemoptysis in two patients with chronic disease, whereas PAE was only performed in three patients with an acute onset of disease. Accordingly, in case of acute onset PAP without bronchial or non-bronchial systemic artery hypertrophy, PAE may be the only treatment option. Therefore, for the effective treatment of PAP, it is important to consider appropriate therapeutic approaches depending on the etiologies and the duration of the underlying disease.

In the current study, pulmonary arteriography was not initially conducted in all cases. Previous studies indicate that PAPs may or may not be detected on pulmonary arteriography [12]. In this respect, the initial assessment of the etiology and evaluation of CT findings play a pivotal role in determining therapeutic approaches for PAPs. In our study, the decision to initially perform pulmonary arteriography was largely based on the CT findings. Accordingly, PAPs were identified, and PAE was possible in 75% of the cases with pulmonary arteriography (15 of 21 patients). In the remaining patients, alternative treatment options such as embolization via systemic-to-pulmonary shunt or BAE/SAE in the corresponding area or medical treatment were adopted according to the clinical presentation. Although a direct approach for pulmonary arteriography was not performed in all cases, previous studies and our results indicate the effectiveness of our strategy [12, 13, 26–30].

This study has some limitations. First, this was a retrospective study with a limited sample size. However, prospective and randomized studies with a large sample size may be difficult, owing to the rarity of this pathology and multiple aspects of the underlying disease. Second, our study did not include treatment with percutaneous transthoracic embolization with direct puncture of the PAP, which can be applicable for PAPs that involve peripheral branches of the pulmonary artery, particularly pleural-based PAPs. Nevertheless, this study has included the largest study population among available reports that explored treatment options for PAPs, and the planning of treatment strategies combining CT and clinical findings will be helpful in guiding therapeutic interventions for PAPs.

In conclusion, PAE is an effective treatment option for PAPs, but empirical embolization via systemic-to-pulmonary shunt can also be an effective alternative treatment for PAPs that were not detected on pulmonary angiography, as well. In addition, medical treatment can be considered for small or isolated PAPs.

## Abbreviations

BAE: Bronchial artery embolization
PAE: Pulmonary artery embolization
PAP: Pulmonary artery pseudoaneurysm
SAE: Non-bronchial systemic artery embolization
